# A Novel Complex Valued Cuckoo Search Algorithm

**DOI:** 10.1155/2013/597803

**Published:** 2013-05-25

**Authors:** Yongquan Zhou, Hongqing Zheng

**Affiliations:** ^1^College of Information Science and Engineering, Guangxi University for Nationalities, Nanning 530006, China; ^2^Guangxi Key Laboratory of Hybrid Computation and IC Design Analysis, Nanning 530006, China

## Abstract

To expand the information of nest individuals, the idea of complex-valued encoding is used in cuckoo search (PCS); the gene of individuals is denoted by plurality, so a diploid swarm is structured by a sequence plurality. The value of independent variables for objective function is determined by modules, and a sign of them is determined by angles. The position of nest is divided into two parts, namely, real part gene and imaginary gene. The updating relation of complex-valued swarm is presented. Six typical functions are tested. The results are compared with cuckoo search based on real-valued encoding; the usefulness of the proposed algorithm is verified.

## 1. Introduction

Recently, a new metaheuristic search algorithm, called Cuckoo Search (CS) [1], has been developed by Yang and Deb (2009). The algorithm is inspired by the reproduction strategy of cuckoos. Because of this method is simple, efficient and optimal random search paths, and successfully applied to practical engineering optimization problems [2]. In term of cuckoo search algorithm, there are many methods to improve its performance; some people study the parameters of cuckoo search algorithm. But these methods are using binary and decimal to encode the bird's nest, individual's information capacity is very limited.

Complex-valued encoding method is already used to express neural network weights [3] and individual genes of evolutionary algorithm [4, 5]; it uses diploid in the expression of individual genes and greatly expands the individual's information capacity. From individual coding method, this paper studies the plural coding performance improvement of cuckoo search algorithm. The value of independent variables for objective function is determined by modules, and the sign of them is determined by angles. The two variables of real and imaginary parts to represent an independent variable, thus nest groups, can enhance the information and tap the individual diversity of the population, reducing the local convergence. We provide a new way for the Cuckoo search algorithm to solve practical problems.

## 2. Cuckoo Search Algorithm

### 2.1. Original CS

CS is a heuristic search algorithm which has been proposed recently by Yang and Deb [1]. The algorithm is inspired by the reproduction strategy of cuckoos. At the most basic level, cuckoos lay their eggs in the nests of other host birds, which may be of different species. The host bird may discover that the eggs are not its own and either destroy the egg or abandon the nest all together. This has resulted in the evolution of cuckoo eggs which mimic the eggs of local host birds. For simplicity in describing the Cuckoo Search, we now use the following three idealized rules:

1. Each Cuckoo lays one egg, which represents a set of solution coordinates, at a time, and dumps it in a random nest.
2. A fraction of the nests containing the best eggs, or solutions, will be carried over to the next generation.
3. The number of nests is fixed and there is a probability that a host can discover an alien egg. If this happens, the host can either discard the egg or the nest and this results in building a new nest in a new location.

Based on these three rules, the basic steps of the Cuckoo Search (CS) can be summarized as the pseudo code shown in Algorithm 1.

When generating new solution *x* ^(*t*+1)^ for, say, cuckoo *i*, a Lévy flight is performed

(1) $$\begin{array}{c} x_{i}^{\left(t+1\right)}=x_{i}^{\left(t\right)}+\partial ⊕\text{Lévy}\left(\beta \right), \end{array}$$

where ∂>0 is the step size which should be related to the scales of the problem of interests. In most cases, we can use ∂ = 1.

The product ⊕ means entry-wise walk during multiplications. Lévy flights essentially provide a random walk while their random steps are drawn from a Lévy Distribution for large steps

(2) $$\begin{array}{c} \text{Lévy}\;\sim \;u=t^{-1-\beta }\;\left(0<\beta <2\right). \end{array}$$

This has an infinite variance with an infinite mean. Here the consecutive jumps/steps of a cuckoo essentially form a random walk process which obeys a power-law step-length distribution with a heavy tail. In addition, a fraction *p*_*a*_ of the worst nests can be abandoned so that new nests can be built at new locations by random walks and mixing. The mixing of the eggs/solutions can be performed by random permutation according to the similarity/difference to the host eggs.

Obviously, the generation of step size s samples is not trivial using Lévy flights. A simple scheme discussed in detail by Yang can be summarized as

(3) $$\begin{array}{c} x_{i}^{\left(t+1\right)}=x_{i}^{\left(t\right)}+\partial ⊕\text{Lévy}\left(\beta \right)\;\sim \;0.01\frac{u}{{\left|v\right|}^{1/\beta }}\left(x_{j}^{\left(t\right)}-x_{i}^{\left(t\right)}\right), \end{array}$$

where *u* and *v* are drawn from normal distributions. That is

(4) $$\begin{array}{c} u\sim N\left(0,\sigma_{u}^{2}\right),\;\;v\sim N\left(0,\sigma_{v}^{2}\right). \end{array}$$

With *σ*_*u*_ = {(Γ(1+*β*)sin(*πβ*/2))/(Γ[(1+*β*)/2]*β*2^(*β*−1)/2^)}^1/*β*^, *σ*_*v*_ = 1. Here Γ is the standard Gamma function [6].

### 2.2. Cuckoo Search Based on Complex-Valued Encoding

Containing the *M*-variable function optimization problem, with *M* complex, corresponding to the *M* complex nest location is recorded as

(5) $$\begin{array}{c} x_{p}=R_{p}+I_{p}j\;p=1,2,\ldots ,M. \end{array}$$

The gene of the nest can be expressed as the diploid and is recorded as (*R*_*p*_, *I*_*p*_); *R*_*p*_, *I*_*p*_ express, respectively, the real and imaginary parts of the variable in (5). So the *i*th nest can be expressed as shown in Table 1.

#### 2.2.1. Initialize the Nest

Assume that the variable interval of function is [*A*_*L*_, *B*_*L*_], *L* = 1,2,…, *M*. Of course, since the interval of the variable is open or half open, half closed, it would not affect the feasibility of the algorithm, such that only for writing convenience. Randomly generating *M*-modules and *M*-angles, the vector of the module and the angle made the following relationship:

(6) $$\begin{array}{c} \rho_{L}=\left[0,\frac{B_{L}-A_{L}}{2}\right],\;\theta_{L}=\left[-2\pi ,2\pi \right],\;L=1,2,\ldots ,M,\\ R_{L}+jI_{L}=\rho_{L}\left(\cos\theta_{L}+j\sin\theta_{L}\right),\;L=1,2,\ldots ,M, \end{array}$$

where *M* real and imaginary parts as shown in Table 2 are assigned to the Bird's Nest, resulting in an initial nest.

#### 2.2.2. The Method of Nest Update

(1) The method of module update is as follows:
  (7) $$\begin{array}{c} \rho_{i}^{\left(t+1\right)}=\rho_{i}^{\left(t\right)}+\partial ⊕L\left(\lambda \right),\;i=1,2,3,\ldots ,n, \end{array}$$

where *ρ*_*i*_^(*t*)^ expresses the *t*th generation value in the *i*th module. The product ⊕ means entry-wise multiplications where ∂>0 is the step size which should be related to the scales of the problem of interest. In most cases, we can use ∂ = 1. *L*(*λ*) ~ *u* = *t* ^−*λ*^, (1 < *λ* ≤ 3). Module vector is updated, if r and >*p*_*a*_ then *ρ*_*i*_^(*t*+1)^ random can be changed, or not changed. The last to retain a good module vector *ρ*_*i*_^(*t*+1)^.

(2) The method of angle update is as follows:
  (8) $$\begin{array}{c} \theta_{i}^{\left(t+1\right)}=\theta_{i}^{\left(t\right)}+\partial ⊕L\left(\lambda \right),\;i=1,2,3,\ldots ,n, \end{array}$$

where *θ*_*i*_^(*t*)^ expresses the *t*th generation value in the *i*th angle. The product ⊕ means entry-wise multiplications, where ∂>0 is the step size which should be related to the scales of the problem of interest. In most cases, we can use ∂ = 1. *L*(*λ*) ~ *u* = *t* ^−*λ*^, (1 < *λ* ≤ 3). Angle vector is updated, if r and >*p*_*a*_ then *θ*_*i*_^(*t*+1)^ random can be changed, or not changed. The last to retain a good angle vector *θ*_*i*_^(*t*+1)^. 

#### 2.2.3. Fitness Calculation

In order to solve the fitness function, plural Bird's Nest must be changed into a real number; the real value of objective function is determined by modules, and sign of them is determined by amplitude angle, specific practices are as follows:

(9) $$\begin{array}{c} \rho_{n}=\sqrt{X_{Rn}^{2}+X_{In}^{2}},\;n=1,2,\ldots ,M\\ \text{R}{\text{V}}_{n}=\rho_{n}\mathrm{sgn}\left(\sin\left(\frac{X_{In}}{\rho_{n}}\right)\right)+\frac{B_{L}+A_{L}}{2},\;n=1,2,\ldots ,M, \end{array}$$

where *ρ*_*n*_ denotes the *n*th dimension module, *X*_*Rn*_, *X*_*In*_ denote the real part and imaginary part of the *n*th dimension; respectively, RV_*n*_ is converted real variable.

## 3. The Basic Steps of PCS

Based on above analysis, the basic steps of complex-valued encoding (PCS) can be summarized as the pseudo code shown in Algorithm 2.

## 4. Simulation Experiment

### 4.1. Design of Experiment

In this section, the performance of the PCS algorithm is extensively investigated by a large number of benchmark optimization problems. All computational experiments are conducted with Matlab7.0 and run on CPU T3100, 1.90 GHZ with 2 GB memory capacity. 

Algorithm parameters are set as follows: because the updates of bird's nest locations are divided into two steps in the complex-valued encoding, there are two update calculations; when the two types of encoding have the same sizes of population, the computational complexity of the complex-valued encoding is approximately two times the one of the real encoding. To compare with the performance of the two methods, the size of plurality population nest is half of the one of real population nest. The size of complex-valued encoding nest is 20, the size of real encoding nest is 40, and the maximum iteration times is 200, *p*_*a*_ = 0.25.

### 4.2. Experimental Results and Analysis

**Figure 1:**
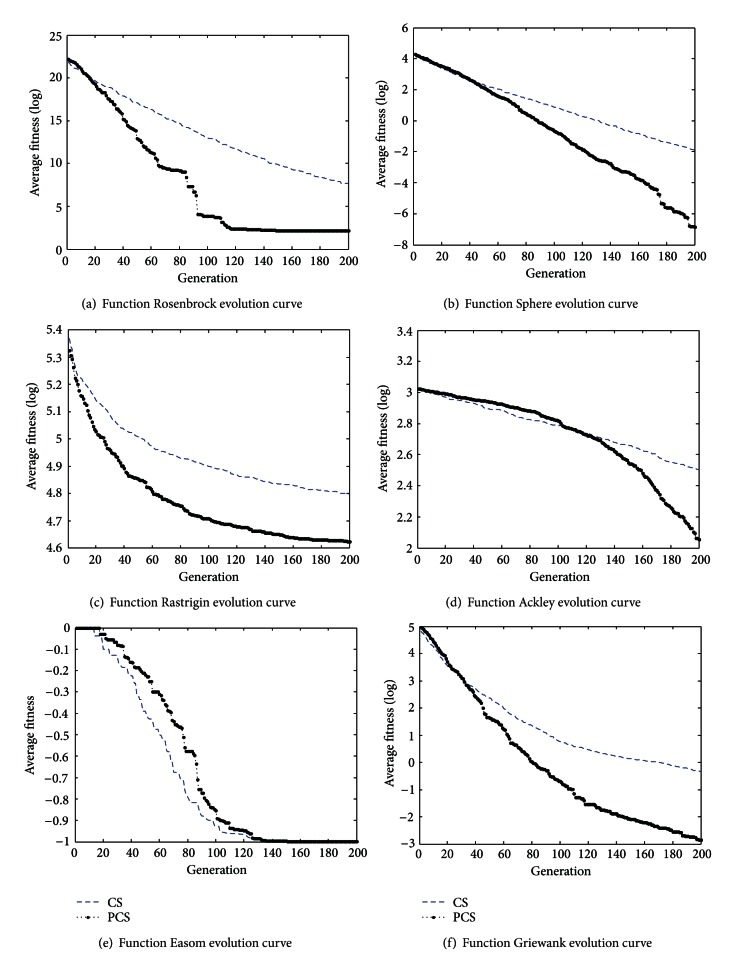
The evolution graph of the average fitness in 20 trails.

In this section, we test on six different functions to verify that the algorithm proposed in this paper is feasible and effective. 20 independent runs are made for the PCS algorithms, and the results obtained by the PCS algorithms are presented in Table 3. From Table 3 we can find that complex-valued encoding method can achieve better fitness than real number coding method. In terms of Rosenbrock function, either best fitness or average fitness, we can see that the precision of PCS is improved 10^2^ and 10^3^ higher than CS, respectively. As far as Sphere function are concerned, the optimal value of PCS reaches the theoretical value; the average fitness is improved 10^3^ higher than CS. About Rastrigin, Ackley function, PCS average value and the optimal ratio of CS are improved, but not obvious. With respect to Easom function, the optimal value of PCS reaches the theoretical value, but CS does not. In terms of Griewank function, the optimal value of PCS can also reach the theoretical value.  Figure 1 shows that this method is better than that real-coded in the convergence rate and convergence precision; this can be explained from the average fitness evolution curves. From the average fitness figures, we can see that the change of average fitness obtained by the complex-valued encoding method is much greater than the one obtained by the real number encoding method, especially in the early evolution. Because the average fitness of the change is bigger, that individual is scattered, not concentrated in one or a few local points. In the iterative process, there is a trend that these points are close to a better location, but this is easy to make the population into the local convergence; therefore maintaining the diversity of the population is very important. Average fitness changes greatly; to some extent, the diversity of the population is better, thus not easy to fall into the local convergence.

## 5. Implement PCS in Determining PID Controller Parameter

In industry process, people generally implement Ziegler-Nichlos rule in determining PID controller parameters; the control effect is generally difficult to meet the requirements of the control system. In this section, we implement PCS in determining PID controller parameters and compare the results with CS and PSO results. The parameters of controller are mapped bird's nest, then optimizing them by the PCS method. In the previous work [7, 8], authors have implemented transfer function from industry

(10) $$\begin{array}{c} G\left(s\right)=\frac{2}{s^{2}+1.5s+2}e^{-0.2s} \end{array}$$

PID Controller can be described as

(11) $$\begin{array}{c} G_{c}\left(s\right)=k_{p}+\frac{k_{i}}{s}+k_{d}s. \end{array}$$

Through adjusting the three parameters, the system satisfies the required performance indicators. The bird's nest is in the three dimensional space encoded; the parameters are set as follows: *k*_*p*_ ∈ [0.01,20], *k*_*i*_ ∈ [0.01,2], *k*_*d*_ ∈ [0.01,2]. The number of nest is 20; the maximum iteration time; is 100. The most crucial step in applying PCS to choose the objective functions *J* that are used to evaluate fitness of each nest; the performance indices are defined as follow [8]:

(12) $$\begin{array}{c} J=\alpha *\int_{0}^{\infty }\left|e\left(t\right)\right|td_{t}+\beta *ts, \end{array}$$

where *e*(*t*) is the error signal in time domain, *ts* is the tuning time, *α*, *β* is weight, which is set as 0.1 and 0.9, respectively.

**Figure 2:**
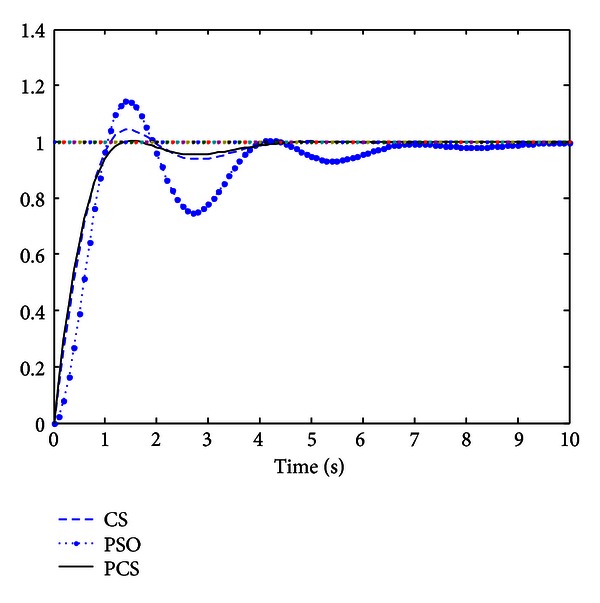
The comparison of unit step response curve.

In the experiments, we implement PSO, CS, and PCS in determining PID controller parameters. The results obtained by the three algorithms are presented in Table 4. Figure 2 is the unit step response curve of control object.

In Figure 2, we can see that the settling time is not so different among all methods; the settling time from short to long is followed by PCS, CS, and PSO. In addition, the overshoot of the three algorithms is (in descending order): PSO, CS, and PCS.

## 6. Conclusions

This paper proposes Cuckoo search based on complex-valued encoding; the individual of Bird's Nest is denoted by plurality, so a diploid swarm is structured by a sequence plurality; the Bird's Nest can express the space dimension much more than the real-coded one. Compared with traditional real-coded, the Bird's Nest also has to contain more information, so that the algorithm improves the search capabilities of the global optimum. To verify the proposed PCS algorithm, also a number of benchmark optimization problems and PID controller parameter tuning are solved using this concept and quite satisfactory results are obtained. CS is the algorithm proposed in the last two years; the theoretical analysis and other applications require further study.

## Figures and Tables

**Algorithm 1:**
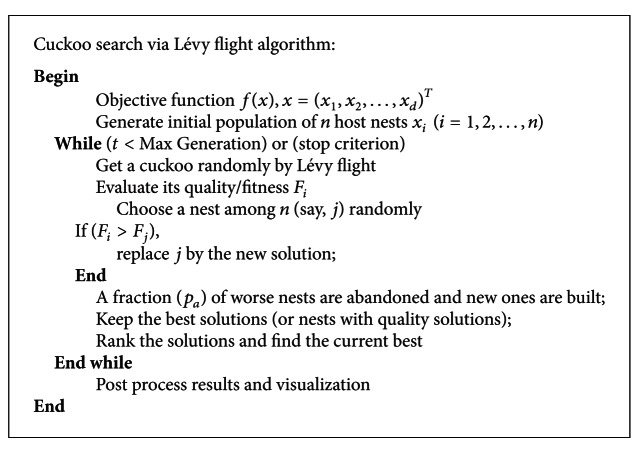
Pseudo code of cuckoo search via Lévy flight algorithm.

**Algorithm 2:**
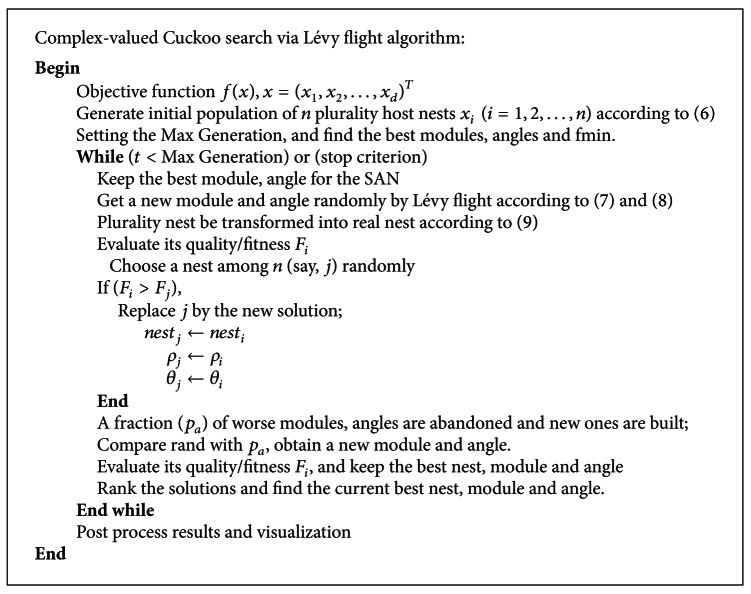
Pseudo code of the plurality cuckoo search (PCS).

**Table 1:** Nest chromosome structure shown.

(*R*_*p*1_, *I*_*p*1_)	(*R*_*p*2_, *I*_*p*2_)	…	(*R*_*pM*_, *I*_*p*M_)

**Table 2:** Test the improved algorithm's benchmark functions.

Functions	Dim	Domain	Theoretical value
*f*(*x*) = ∑_*i*=1_^*d*−1^[(1−*x*_*i*_)^2^ + 100(*x*_*i*+1_−*x*_*i*_^2^)^2^]	10	[−100,100]	0
*f*(*x*) = ∑_*i*=1_^*d*^ *x*_*i*_^2^	15	[5.12,5.12]	0
*f*(*x*) = 10*d* + ∑_*i*=1_^*d*^[*x*_*i*_^2^ − 10cos⁡(2π*x*_*i*_)]	20	[5.12,5.12]	0
$f(x)=-20\exp\left[-0.2\sqrt{\left(1/d\right)\sum_{i=1}^{d}x_{i}^{2}}\right]-\exp\left[\left(1/d\right)\sum_{i=1}^{d}\cos(2\pi x_{i})\right]+20+e$	30	[−32.768,32.768]	0
*f*(*x*) = −cos⁡(*x*)cos⁡(*y*)exp⁡[−(*x*−π)^2^ − (*y*−π)^2^]	2	[100,100]	−1
$f(x)=1+\left(1/4000\right)\sum_{i=1}^{d}x_{i}^{2}-\prod_{i=1}^{d}\cos\left(x_{i}/\sqrt{i}\right)$	10	[−600,600]	0

**Table 3:** The results of experiment in running 20 times.

Functions	Algorithm	Best	Worst	Mean	Variance
Rosenbrock	CS	671.2474	4.8400*e* + 003	2.1090*e* + 003	1.0339*e* + 006
	**PCS**	**7.9206**	**8.9835**	**8.3874**	**0.1028**
Sphere	CS	0.0648	0.1723	0.1141	0.0012
	**PCS**	**0**	**0.0088**	8.9793**e** − 004	4.6582**e** − 006
Rastrigin	CS	112.8084	128.5006	120.8996	20.0181
	**PCS**	**100**	**104.0008**	**100.8662**	**1.6589**
Ackley	CS	10.7296	13.7988	12.3351	0.5920
	**PCS**	**0.0052**	**13.0287**	**7.7304**	**13.5185**
Easom	CS	−1	−0.9992	−0.9998	4.1112*e* − 008
	**PCS**	−1	−0.9998	−1	3.1784**e** − 009
Griewank	CS	0.4814	0.9043	0.7230	0.0111
	**PCS**	**0**	**0.1129**	**0.0256**	**0.0017**

**Table 4:** PID Controller tuning parameters.

Tuning method	*k*_*p*_	*k*_*i*_	*k*_*d*_
PSO	0.0100	2.0000	1.0518
CS	0.6491	2.0000	2.0000
PCS	0.8096	1.9738	1.9995
